# Two Outbreaks of Pigeon Paramyxovirus 1 With High Mortality in Captive Pigeons (*Columbia livia*) in Denmark, 2022–2023

**DOI:** 10.1155/tbed/5629889

**Published:** 2025-09-29

**Authors:** Karen Martiny, Audra-Lynne D. Schlachter, Tim K. Jensen, Fabian Z. X. Lean, Alejandro Núñez, Anne Sofie Hammer, Christian Grund, Dirk Höper, Solvej Ø. Breum, Jens P. Christensen, Lars E. Larsen, Charlotte K. Hjulsager

**Affiliations:** ^1^Department of Veterinary and Animal Sciences, University of Copenhagen, Dyrlægevej 88, Frederiksberg C 1870, Denmark; ^2^Department of Pathology and Animal Sciences, Animal and Plant Health Agency (APHA-Weybridge), New Haw, Addlestone, UK; ^3^Department of Veterinary and Animal Sciences, University of Copenhagen, Ridebanevej 3, Frederiksberg C 1870, Denmark; ^4^Department of Pathobiology and Population Sciences, The Royal Veterinary College, Hawkshead Lane, North Mymms, Hatfield AL9 7TA, UK; ^5^Institute of Diagnostic Virology, Friedrich-Loeffler-Institut, Greifswald 17493, Germany; ^6^Department of Virology and Microbiological Preparedness, Statens Serum Institut, Artillerivej 5, Copenhagen S 2300, Denmark

**Keywords:** avian paramyxovirus 1, newcastle disease, orthoavulavirus javaense, outbreak, pigeon paramyxovirus 1, pigeons, virulence

## Abstract

This study describes the first outbreaks with virulent avian paramyxovirus 1 (APMV-1) since 2005 in Denmark. Both outbreaks were caused by pigeon specific variants, denoted pigeon paramyxovirus 1 (PPMV-1). The first outbreak was in June 2022 and affected captive pigeons near Næstved, in southeast Denmark, where 1053 captive birds were housed, and hereof 851 pigeons. A second and separate outbreak occurred in June 2023 in Aalborg, northwest Denmark, involving 1851 captive birds, of which 40 were pigeons. In both outbreaks, pigeons were predominantly affected, characterised by high mortality, and presented with neurological signs, along with thin-shelled eggs. Pathological and virological assessment revealed multi-systemic infections in pigeons, including neuronal and vascular endothelial tropism. Chickens were affected only in the 2022 outbreak, with reported extended hatching periods and chicks dead at hatching, and with no apparent lesions detected at both macro- and microscopic investigations. Fusion protein (F) gene sequence classified the 2022 virus isolate as genotype VI.2.1.1.2.2 and the 2023 virus as genotype XXI.1.1, with polybasic cleavage sites ^112^RRQKRF^117^ (2022) and ^112^KRQKRF^117^ (2023). However, mean death time (MDT) tests categorised both virus isolates as mesogenic, and an intracerebral pathogenicity index (ICPI) test of the 2022 virus isolate showed an ICPI index of 0.65, categorising the virus as lentogenic. This is the first report of PPMV-1 isolates with polybasic cleavage site and associated mortality in captive pigeons in Denmark. The sudden resurgence of outbreaks in Denmark after nearly two decades without similar incidents highlight the potential threat posed by circulating viruses in wild birds, such as feral pigeons, and emphasise the importance of surveillance in wild bird populations for improved risk recognition and early detection of emerging threats.

## 1. Introduction

Avian paramyxovirus 1 (APMV-1), is classified as *Orthoavulavirus javaense* within the genus *Orthoavulavirus* in the family Paramyxoviridae. APMV-1 can infect over 200 bird species, with clinical outcomes ranging from subclinical infection to systemic disease with high mortality, depending on both viral and host factors [1]. Virulent strains of APMV-1 are the causative agent of Newcastle disease (ND), an economically significant and often severe fatal disease in the poultry sector [2–4].

APMV-1 can be categorised into two classes based on genome length and fusion protein (F) gene sequence: class I, comprising a single genotype (1), and class II, comprising 21 genotypes (I–XXI) [5]. Strains isolated from *Columbiformes* (pigeons and doves) predominantly cluster within genotypes VI and XXI, reflecting their adaptation to these hosts, and are referred to as pigeon paramyxovirus 1 (PPMV-1) [5, 6].

PPMV-1 emerged in the Middle East in the 1970s in racing pigeons, and spread globally since the 1980s [7, 8]. Today, PPMV-1 remains endemic in pigeons in many regions, including Europe, and recent years have seen a resurgence in outbreaks. While PPMV-1 affected pigeons commonly presents with neurological signs, subclinical infection is also frequent [9]. Although occasional spillover into poultry has occurred, outbreaks in chickens remain sporadic and are typically limited to isolated flocks [10–13].

APMV-1 strains are biologically pathotyped by intracerebral pathogenicity index (ICPI) scores and mean death time (MDT) tests in chickens, which categorise them into lentogenic, mesogenic, or velogenic pathotypes [4]. APMV-1 can also be pathotyped by molecular methods based on the nucleotide sequence encoding the F protein cleavage site. Virulent strains possess a polybasic cleavage site, which can be cleaved by ubiquitous proteases, enabling systemic infection. In contrast, avirulent strains have a monobasic cleavage site, which can only be cleaved by extracellular proteases, confining the infection to the respiratory and gastrointestinal tracts. According to the World Animal Health Organisation (WOAH), APMV-1 is classified as virulent if it has a polybasic cleavage site or an ICPI score above 0.7. The designation ND virus (NDV) is, therefore, commonly and predominantly used for such virulent strains.

In Denmark, ND is a notifiable disease, and mandatory vaccination of chickens has been enforced since 2004 [14]. The last outbreak occurred in 2005 during the initial implementation of vaccination in a flock that had not yet been vaccinated. For pigeons, vaccination is required only under specific circumstances, such as for certain races and exhibitions. In 2022 and 2023, Denmark experienced the first APMV-1 outbreaks since 2005, both caused by PPMV-1. These outbreaks occurred in Danish allotment house communities housing large populations of birds, including pigeons (*Columbia livia*) and chickens (*Gallus gallus domesticus*). This study describes the two outbreaks, while focusing on the genetic characterisation and pathotyping of the viruses and pathological, histopathological and immunohistochemical (IHC) assessment of the affected birds.

## 2. Materials and Methods

### 2.1. Case Description and Sampling, Outbreak June 2022

On 31 May 2022, a veterinarian contacted the Danish veterinary authorities, the Danish Veterinary and Food Administration (DVFA), with a suspicion of the notifiable diseases ND, avian influenza (AI) and ornithosis (synonymous with Psittacosis caused by *Chlamydia psittaci*) in an allotment house community in Stenbæksholm and Stenstrup, near Næstved in the southeast part of Denmark. The allotment house community had 23 owners and housed 1053 captive birds, including 851 pigeons, 170 chickens, 14 canaries, nine zebra finches, seven cockatiels and two parrots. Of these, 100 pigeons were kept at the residence in Stenstrup, owned by one of the allotment house members, while the remaining birds were housed in the Stenbæksholm allotment house community. The pigeons included both fancy pigeons and racing pigeons. The DVFA, in collaboration with the Danish national reference laboratory for AI and ND at Statens Serum Institut (SSI), coordinated the investigation.

Birds in the allotment house community had shown clinical signs of disease for an extended period (weeks to months, according to owners), which had worsened by the end of May. The disease primarily affected the pigeons, and clinical signs observed in the affected birds included neurological signs such as tremors and torticollis, facial swelling, anorexia, decreased egg production, thin-shelled eggs, diarrhoea and increased mortality. The only observations of disease in chickens were an owner reporting prolonged hatching of eggs with chicks dying at hatching, and a chicken being euthanized due to undisclosed clinical signs. Owners also noted that some pigeons appeared to recover from disease only to become sick again. Mortality in pigeons varied between houses, with some owners reporting rates ranging from 25% to 100%, with the highest rates observed in young pigeons. Some owners reported no clinical signs and no mortality, indicating that not all allotment houses were affected by disease. PPMV-1 vaccination coverage varied among the birds because of different ownership practices, but the disease primarily affected unvaccinated pigeons, according to owner reports.

Since pigeons was the species predominantly affected, samples to investigate the suspicion were initially collected from this species. For APMV-1 and AI virus (AIV) analysis, four pools of five oropharyngeal swabs and four pools of five cloacal swabs were collected from live pigeons and one pool of oropharyngeal swabs and one pool of cloacal swabs pool was collected from five dead pigeons. Swabs were pooled in virus transport media (SSI Diagnostica, Hillerød, Denmark). Separate swab samples were collected in 0.9% saline solution for *C. psittaci* analysis, consisting of 10 pools of oropharyngeal swabs from three pigeons each and 10 pools of cloacal swabs from three pigeons each. The clinical status of these pigeons was undisclosed. All the swab pools were collected on 31 May. Outbreak with ND, virulent APMV-1, was declared on 2 June 2022 by the DVFA.

Upon confirmation of virulent AMPV-1, nine dead pigeons (four male and five female), one culled female pigeon and one female chicken culled with clinical signs, were collected from four allotment houses for pathological, histopathological and IHC examination. For histopathology and IHC, the following tissues were collected: brain, heart, lung, trachea, liver, pancreas, small intestine, kidney, spleen, ovary, proventriculus and salpinx. Cloacal swabs and tissues including pancreas, kidney, duodenum, spleen, caecal tonsils, cloaca, trachea, lung, heart, liver and brain were collected for additional APMV-1 virological examinations and blood samples from 11 pigeons were collected on 3 June.

Due to the close proximity of the allotment house community in Stenbæksholm and the private residence in Stenstrup, the two locations were treated as a single epidemiological unit. All birds in the allotment house community and the private residence were culled using carbon monoxide, and both locations were subsequently disinfected. A 3 km protection zone and a 10 km surveillance zone were established around the affected sites. There was no evidence of disease spread within the zones, including to four contact holdings, one of which was located outside the 10 km surveillance zone.

### 2.2. Case Description and Sampling, Outbreak June 2023

A second outbreak occurred in June 2023 in an allotment house community in Aalborg, in the northwest part of Denmark. A suspicion of ND and AI was reported to the DVFA and the investigation was coordinated in collaboration with SSI.

The allotment house community consisted of 37 owners and housed 1896 birds, including 40 pigeons. The premises also housed ornamental chickens and various other ornamental birds, such as canaries and parrots. Unlike the 2022 outbreak, where both pigeons and chickens exhibited clinical signs, pigeons were apparently the only birds affected in this outbreak, with 30 out of 40 succumbing to disease. Affected pigeons displayed clinical signs consistent with the previous outbreak, including neurological signs, anorexia, decreased egg production, thin-shelled eggs, diarrhoea and increased mortality. The vaccination status of the birds remained unknown.

Samples for AI and ND virus analysis to investigate the suspicion were collected from pigeons on 19 June 2023, as these were the primarily affected species. Oropharyngeal and cloacal samples were collected as follows: three pools of four to five swabs from symptomatic pigeons, one pool of five swabs from asymptomatic pigeons, and one pool of two swabs from dead pigeons; swabs were pooled in virus transport media (SSI Diagnostica). Additionally, two dead pigeons (one male and one female) were submitted for analysis. For histopathology and IHC, brain, trachea, lung and intestine were collected. Additionally, these tissues, along with caecal tonsils and tracheal and cloacal swabs, were tested for APMV-1 by rRT-PCR. Outbreak with ND, virulent APMV-1, was declared by DVFA on 21 June 2023.

On 22 June, 10 chickens (one male and nine females; one dead and nine culled) housed in close contact with the affected pigeons were collected for pathological and virological investigations. Two pools of five oropharyngeal swabs and two pools of five cloacal swabs were collected from the chickens.

Following the confirmation of virulent APMV-1, all birds in the allotment house community were culled using carbon monoxide, the entire allotment house community was disinfected and protective measures were implemented, including establishment of a 3 km protection zone and a 10 km surveillance zone around the premises. There was no evidence of spread of disease within the zones, including to six contact holdings and nearby farms.

**Figure 1:**
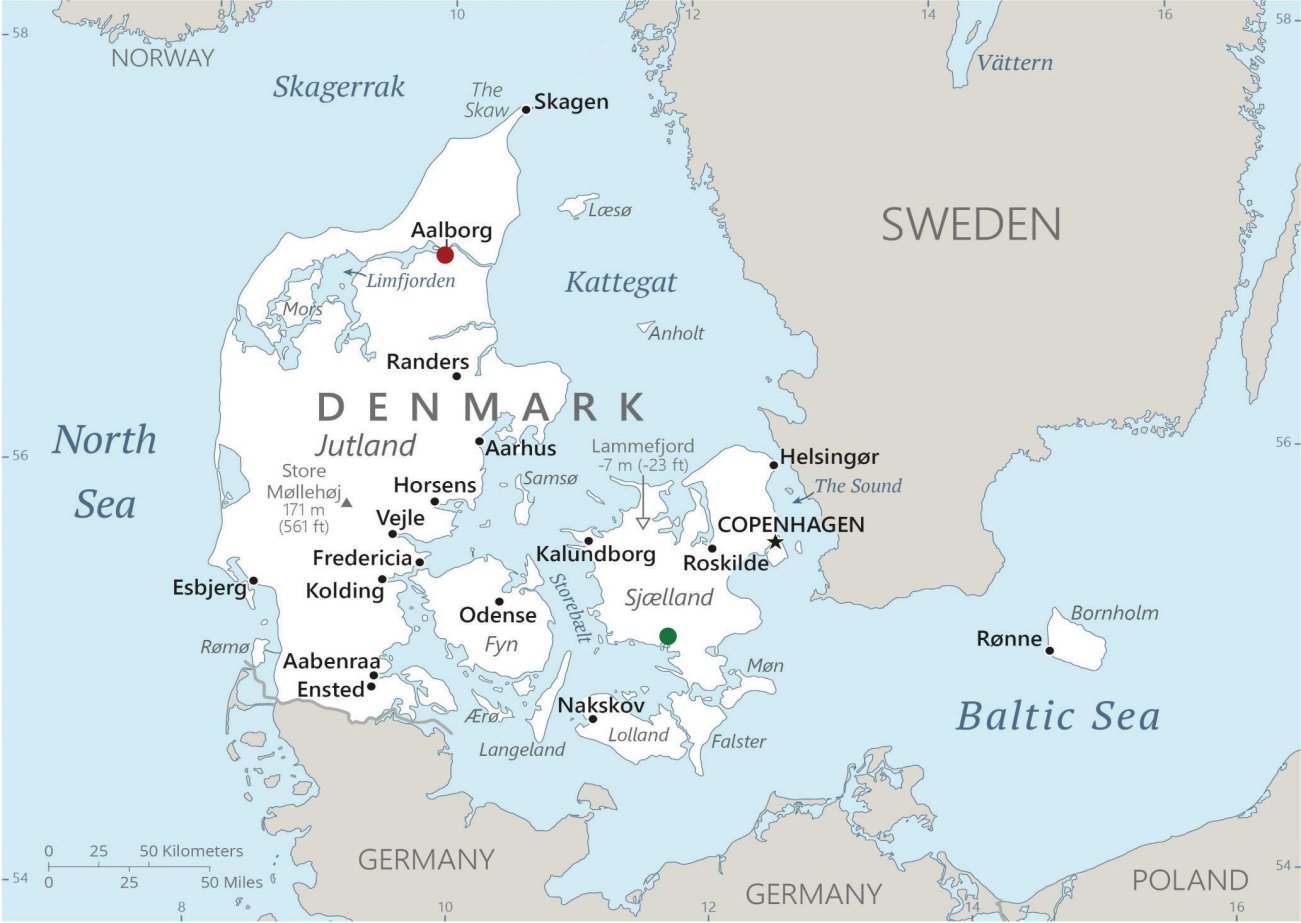
The locations of the 2022 outbreak near Næstved (Stenbæksholm and Stenstrup) (green) and the 2023 outbreak in Aalborg (red) in Denmark.

A map illustrating the locations of the 2022 and 2023 outbreaks was created using a map of Denmark sourced from The World Factbook [15]. The map was edited to include markers indicating the outbreak locations (Figure 1).

### 2.3. Gross Pathology, Histopathology and Immunohistochemistry

Systematic gross examinations were performed on the pigeons and chickens. For histopathology, tissue samples were fixed in 10% neutral-buffered formalin and processed and embedded in paraffin. A 4 µm tissue sections were stained with haematoxylin and eosin (H&E) for histopathology and serial sections were prepared and stained using mouse monoclonal anti-NDV nucleoprotein (NP) Q50 (Australian Centre for Disease Preparedness, Australia), as described in Mahmood et al. [16]. Concentration matched isotype controls were performed using mouse IgG (Vector Laboratories, UK). IHC labelling was assessed using a semi-quantitative scoring system to describe immunolabelling, where 0 = absent, 1 = rare, 2 = low numbers, 3 = moderate numbers and 4 = abundant (coalescing to diffuse) immunolabelled cells.

### 2.4. Pathogen Detection

Samples were tested for AIV and NDV with methods in concordance with recommendations by the European Reference Laboratory for AI and ND (EURL). Swabs were collected in virus transport media (SSI Diagnostica). To elute material from the swabs they were shaken prior to viral RNA extraction for 30 min. Subsequently, 200 µL of swab material was mixed with 400 µL Buffer RLT containing 1% β-mercaptoethanol. Tissue samples were homogenised on a TissueLyzer II (QIAGEN) with 5 mm stainless steel beads for 3 min at 30 Hz mixing 70 mg of tissue in 1400 µL Buffer RLT containing 1% β-mercaptoethanol. The homogenates were centrifuged at 12,000 rpm for 3 min, and 600 µL of supernatant was used for RNA extraction. For both swabs and tissue-homogenates, RNA was extracted with the RNeasy Mini Kit (QIAGEN, Aarhus, Denmark) and the large sample protocol on the QIAcube extraction robot (QIAGEN) with an elution volume of 100 µL. Test for APMV-1 was performed using rRT-PCR assays targeting the L-protein [17] and M-protein [18], with slight modifications as previously described [19]. For virulence determination, F gene RT-PCR was performed according to EURL SOP VIR 151 based on [20, 21], and the PCR product was Sanger sequenced with the PCR primers and the sequence was evaluated as virulent or avirulent according to the WOAH manual [22]. Detection of AIV was performed by rRT-PCR assays targeting the matrix and haemagglutinin genes as previously described [23]. Additionally, samples from the 2022 outbreak were screened for *Chlamydia psittaci* [24].

### 2.5. Virus Isolation

Oropharyngeal and cloacal swab material was sterile filtered (0.45 µm filter (Sartorius, Göttingen, Germany)) and inoculated into the allantoic cavity of 9–11-day-old specific-pathogen-free (SPF) embryonated chicken eggs (ECEs) (Valo Biomedia, Osterholz-Scharmbeck, Germany), and incubated at 37°C for up to 6 days and candled daily. All isolates were passaged twice, and the harvested allantoic fluids were tested for hemagglutination (HA) activity by rapid HA test after each passage by mixing approximately 10 µL of harvested allantoic fluid with an equal volume of 5% red blood cells (RBCs) derived from SPF chickens (Håtunalab, Bro, Sweden).

### 2.6. HA Inhibition (HI) Test

Harvested allantoic fluids testing positive in the rapid HA test were tested for HI activity according to the WOAH protocol using 4 HA units of virus, and APMV-1 genotype II (LaSota) and PPMV-1 genotype VI.1 (APMV1/dove/Italy/4400/2000) reference polyclonal antisera provided by the EURL (IZSVe), as well as the monoclonal antibodies U85, 161/617 and 7D4 [25, 26]. Blood collected from 11 pigeons from the 2022 outbreak was tested by HI test against the isolated outbreak virus (P/DK/04977–10/2022), and against reference antigens APMV-1 (LaSota) and PPMV-1 provided by the EURL. HI titres were expressed as the highest dilution causing complete agglutination and titres above 8 were considered positive.

### 2.7. MDT and ICPI Test

The pathogenicity of both outbreak viruses was characterised by determining the MDT. A series of 10-fold dilutions (10^−1^–10^−9^) of 0.1 mL virus isolate was inoculated in the allantoic cavity of 9-day-old SPF ECE. Five eggs were used per virus dilution and five control eggs were inoculated with sterile PBS. The eggs were incubated at 37°C for 7 days and candled twice daily at 10 am and 4 pm. The highest dilution where all embryos died was determined as the mean lethal dose (MDL) and the MDT was determined as the average time at which all eggs died. The MDT pathogenicity criteria were categorised as follows: velogenic viruses having a MDT below 60 h (h), mesogenic between 60 and 90 h and lentogenic above 90 h [27, 28].

The pathogenicity of the 2022 outbreak isolate was also determined by ICPI test at the Friedrich-Loeffler-Institut (FLI) according to WOAH recommendations [22] in agreement with the German animal health law (registration 7221.3-2-009/19). Briefly, 0.05 mL of a 10^−1^ dilution of infectious allantoic fluid with a HA titre of 128 was injected intracerebrally in 10-day-old SPF chickens. For 8 days, the chickens were observed daily and scored according to level of sickness (0 if normal, 1 if sick, 2 if dead). The ICPI index was calculated as the mean score per bird per observation. Isolates with ICPI scores below 0.7 are considered lentogenic, those with scores between 0.7 and 1.5 are considered mesogenic, and scores above 1.5 indicate velogenic isolates [4].

### 2.8. Fusion Gene Sequencing and Phylogenetic Analysis

Sequencing of the complete F gene was performed as previously described [19]. Briefly, the complete F gene was amplified by RT-PCR using the 7-S and 9-A primers [29] and the SuperScript III One-Step RT-PCR System with Platinum Taq High Fidelity Kit (Invitrogen, Thermo Fischer Scientific, Roskilde, Denmark). The PCR products were sequenced using the Nextera XT DNA Library Preparation Kit (Illumina, Copenhagen, Denmark) on the Illumina MiSeq System (Illumina). Sequencing reads were processed using CLC Genomics Workbench version 22 (QIAGEN, Aarhus, Denmark). Paired reads were de novo assembled, and matching reference sequences were selected using Basic Local Alignment Search Tool (BLAST) in the GenBank database. Consensus sequences were generated by mapping reads to the selected reference sequences.

For phylogenetic analysis, the consensus sequences were trimmed to contain only the F gene and aligned with a curated pilot collection of Class II genotypes [5] and with a collection of genotype VI and XXI sequences along with best-matching sequences identified through BLAST against the GenBank nucleotide database for each outbreak virus (Tables S1 and S2). Phylogenetic trees were constructed in IQ-TREE version 2.0.3 [30] by maximum likelihood analysis (1000 bootstrap replicates). Model testing was done using the software jModelTest v.2.1.10 [31, 32] and the generel time reversible (GTR) model with a discrete gamma distribution (+G) and allowing for invariable sites (+I) was chosen as the best fitted model. Phylogenetic trees were visualised in FigTree v1.4.4 [33]. Pairwise nucleotide distances between outbreak isolates and their closest relatives were calculated from aligned F gene sequences with CLC Genomics Workbench version 22 (QIAGEN, Aarhus, Denmark) to assess genetic relatedness.

### 2.9. Whole Genome Sequencing and Bioinformatics Analysis

The complete genomes of the two outbreak isolates were sequenced using a next-generation sequencing (NGS) metagenomics method [34]. The isolates were assigned the library ID lib05964 (2022 isolate) and ID lib06972 (2023 isolate). The workflow was previously described in detail [35]. Briefly, RNA was extracted from infectious allantoic fluid with the RNAdvance Tissue Kit (Beckman Coulter, Krefeld, Germany) on the KingFisher Flex instrument (Thermo Fischer Scientific), and quantified on a Nanodrop ND1000 (PeqLab, Erlangen, Germany). RNA was converted into cDNA by using a SuperScript IV First-Strand Synthesis System (Life Technologies, California, USA) and the NEBNext Ultra II Non-directional RNA Second Strand Synthesis Module (New England Biolabs Ltd., Massachusetts, USA), and fragmented with a Covaris M220 sonicator (Covaris, Brighton, United Kingdom). Libraries were built with the GeneRead DNA Library L Core Kit (Qiagen) and an Ion Xpress Barcode Adapter (Life Technologies), and quantified with the QIAseq Library Quant Assay Kit (Qiagen) and sequenced on an Ion Torrent S5 XL system (Thermo Fisher Scientific) on an Ion 530 chip in 400 Bp mode. The reads were analysed with RIEMS [36] for taxonomic binning, Diamond blastx [37] for APMV-1 identification and references and Newbler v 3.0 (Roche) for assembly and mapping.

The complete coding sequences (CDS) from the present study have been uploaded to the GenBank database. The 2022 outbreak virus was designated as PPMV-1/pigeon/Denmark/04977-10/2022 (P/DK/04977-10/2022) with the accession number PV260999 and the 2023 outbreak virus was designated as PPMV-1/pigeon/Denmark/05038-4/2023 (P/DK/05038-4/2023) with the accession number PV261000.

### 2.10. Analysis of Amino Acid Changes and Host-Specific Adaptations

Amino acid changes associated with virulence, antigenic sites and host adaptation were analysed in the genomes of the outbreak causing viruses (Table S9). The analysis incorporated previously reported changes from multiple studies [38–45]. Host-specific sites identified in Huang et al. [6] and Dortmans et al. [40] were also included to investigate potential adaptations to chickens.

## 3. Results

### 3.1. Outbreak June 2022

#### 3.1.1. Pathogen Detection

APMV-1 was detected in eight out of 10 swab pools collected from pigeons in the allotment house community in Stenbæksholm and Stenstrup, in Southeast Denmark (Figure 1). Sequencing confirmed a polybasic F protein cleavage site, classifying the virus as virulent. *Chlamydia psittaci* was detected in two out of 20 swab pools. AIV was not detected in the samples. All PCR results are listed in Tables S3 and S4. APMV-1 antibodies were detected by HI-test in blood samples from three out of 11 pigeons (Table S5). APMV-1 was isolated in ECE from four pools; one pool of oropharyngeal swabs and one pool of cloacal swabs collected from live pigeons, and one pool of oropharyngeal swabs and one pool of cloacal swabs collected from dead pigeons. The virus isolated from the pool of cloacal swabs collected from dead pigeons was designated PPMV-1/pigeon/Denmark/04977-10/2022 (P/DK/04977-10/2022) and selected for further virologic characterisation.

#### 3.1.2. Gross Pathology

Ten pigeons and one chicken from four different allotment houses were necropsied. The pigeons (six females and four males) were all adults, and presented in poor (*n* = 1), to very poor (*n* = 9) body condition. The plumage was generally unremarkable but faecal soiling was present around the vent in five birds. Periocular hyperaemia and oedema were noted in three pigeons. Pulmonary congestion was observed in seven birds, occasionally accompanied by haemorrhage, and in a single bird multiple pale yellow foci (abscesses/necrosis) were seen in the lungs. Congestion of the liver was evident in nine birds, with few yellow white foci (abscesses/necrosis) visible in the hepatic parenchyma of one bird. The kidneys appeared swollen, pale and friable. Proventriculus and gizzards contained little food material and intestinal contents were sparse. The remaining organs examined, including brain and heart, were unremarkable. No gross abnormalities were seen in the chicken.

#### 3.1.3. Histopathology and IHC

**Figure 2:**
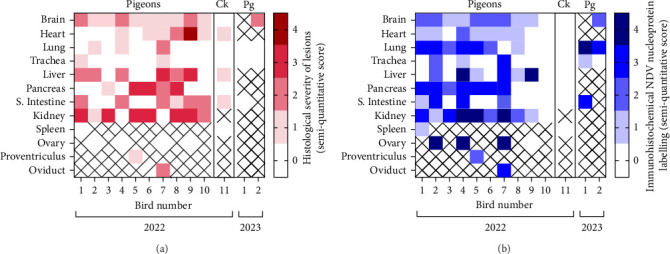
Histopathological and immunohistochemical characterisation of lesions and NDV nucleoprotein distribution in 10 pigeons and a chicken (Ck) in outbreaks in 2022, and two pigeons (Pg) in 2023. Categorical heat maps denote (a) the severity of histopathological changes and (b) the abundance of viral antigen. For the histopathology heat map, 0 = absent; 1 = minimal; 2 = mild; 3 = moderate; 4 = marked. Lesions not associated with NDV infection were excluded from the score. Immunohistochemistry scoring is defined as: 0 = absent, 1 = rare, 2 = low numbers, 3 = moderate numbers and 4 = abundant immunolabelled cells. A cross (‘x') in the heat map indicates samples were not available. S. intestine refers to small intestine.

**Figure 3:**
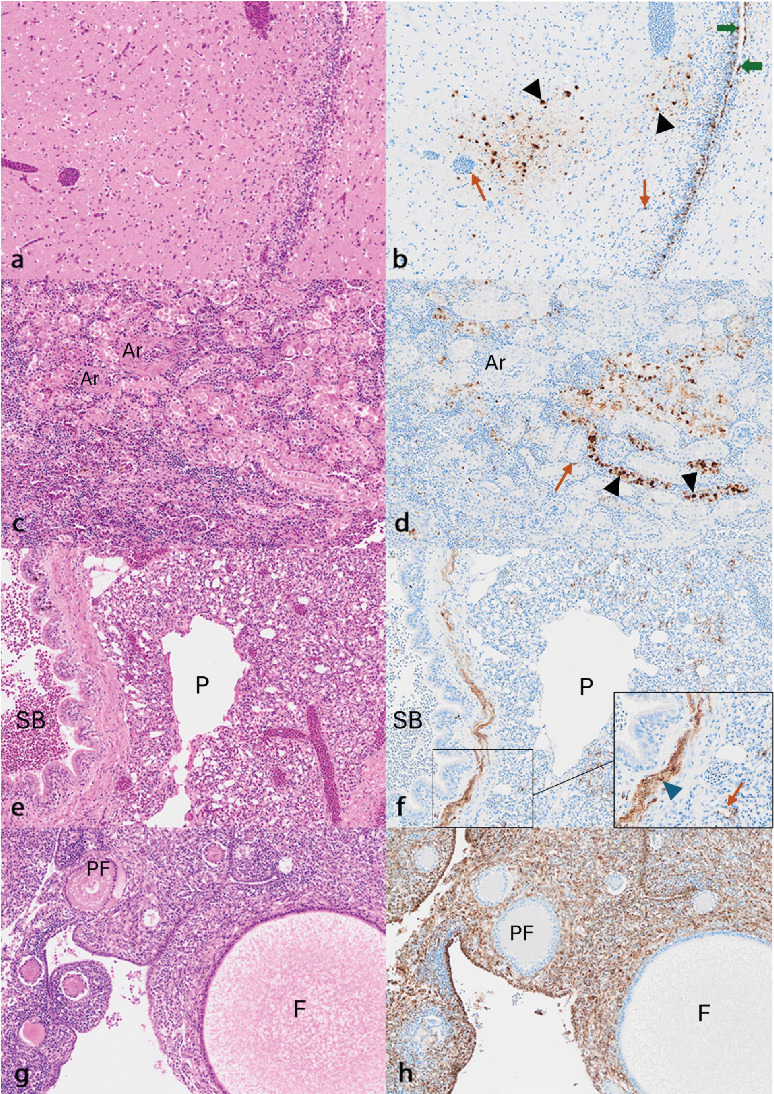
Histopathological and viral immunohistochemical correlates in PPMV-1 infected pigeons. (a) Mild gliosis in the brain, with (b) viral antigen present in neurones (black arrowheads), ependymal cells (blue arrows) and endothelium (orange arrows); (c) moderate lymphoplasmacytic tubulointerstitial nephritis, with (d) immunolabelling in the tubular epithelium (black arrowheads) and endothelium (orange arrows); (e) minimal diffuse pulmonary congestion with (f) virus antigen within endothelium of the air capillaries (inset; orange arrow) and in fibroblasts beneath the bronchiolar epithelium (inset; blue arrowhead); (g) normal follicular activity with multiple follicles in varying stages of development and (h) diffuse immunolabelling within fibroblasts of the supporting cortical stroma. Original magnification for all images 20x, (h) inset x40. H&E and NDV nucleoprotein antibody immunolabeled images represent serial sections. Ar, arteriole; F, developing follicles; P, parabronchus; PF, primary follicles; SB, secondary bronchus.

Multiple tissues from the necropsied 10 pigeons and one chicken were assessed by histopathology and viral IHC (Figures 2 and 3).

In the pigeons, lesions were most commonly present in the brain, where minimal to mild, multifocal, non-suppurative encephalitis (Figure 3a), characterised by gliosis, occasional neuronal degeneration and minimal mononuclear perivascular cuffing was seen in all pigeons (*n* = 10/10) assessed. Meningitis was also observed in a single bird. Infrequent viral antigen was detected in 8/10 birds mainly within the endothelium, but also within ependymal cells, epithelium of the choroid plexus and infrequently in neurones (Figure 3b). In the single bird with meningitis, antigen was present in the meninges surrounding the cerebellum and within Virchow–Robins spaces, in meningeal cells and vascular endothelium. In the kidneys, a moderate multifocal lymphoplasmacytic tubulointerstitial nephritis (Figure 3c) was identified in 8/10 birds. Abundant viral antigen was detected both associated with (*n* = 7/10) and without (*n* = 2/10) lesions, predominantly in tubular epithelial cells and within cellular debris in the tubular lumen, as well as in the interstitial endothelium (Figure 3d). In the remaining bird, mild multifocal chronic-active interstitial nephritis with mild multifocal lymphoid hyperplasia was observed but no antigen was identified.

In the lungs, diffuse pulmonary congestion (*n* = 6/10; Figure 3e), minimal lymphocytic bronchiolitis (*n* = 2/10), and pyogranulomatous pneumonia with intra-lesional bacterial colonies and fungal hyphae (*n* = 4/10) were seen. Viral antigen was found often without associated lesions in 8/10 birds, within endothelium, bronchial epithelium, and occasionally within fibroblasts and macrophages (Figure 3f). In the trachea there was minimal multifocal lymphocytic tracheitis in one bird, with colocalisation of virus antigen in the endothelium and epithelium. In the heart, minimal, focal to multifocal lymphocytic epicarditis (*n* = 5/10) was seen, with infrequent viral antigen observed in the mesothelium and endothelium (*n* = 4/10), and occasionally, myocardium (*n* = 1/10). In addition, marked multifocal acute fibrinonecrotizing myocarditis and epicarditis were detected in a single bird with no associated immunolabelling. The spleen was available for examination in 2/11 birds, but no overt lymphocytolysis or lymphoid necrosis was detected, and labelling was rarely detected in the endothelium.

In the pancreas, exocrine pancreatic necrosis (*n* = 3/10) or minimal to moderate multifocal mononuclear pancreatitis (*n* = 3/10) was observed. Viral antigen was detected in the acinar epithelium and interstitial endothelium (*n* = 7/10), typically (*n* = 5/7), but not consistently (*n* = 2/7), associated with histopathological lesions. In the liver, variable lesions were present, but those positive for viral antigen (*n* = 5/10) demonstrated either no overt lesions (*n* = 1/5), mild multifocal hepatocellular degeneration (*n* = 2/5), or moderate multifocal mononuclear hepatitis (*n* = 1/5). Virus antigen was detected mainly in endothelium, Kupffer cells and occasionally in hepatocytes. One of these five birds had a severe multifocal pyogranulomatous hepatitis associated with intralesional nematodes, and moderate to severe diffuse chronic lymphoplasmacytic cholangitis. In this case, abundant viral antigen was detected in fibroblasts and endothelium of the capsule surrounding the pyogranulomatous lesions. Other incidental non-specific findings include mild multifocal lymphocytic hepatitis and extramedullary haematopoiesis (*n* = 4/10).

Proventriculus was available for examination in one bird, in which focal glandular dilation with epithelial attenuation and abundant intraluminal proteinaceous exudate was observed, with viral antigen colocalised in glandular epithelium and endothelium. In the small intestine, mild diffuse lymphoplasmacytic enteritis was often seen in 6/10 birds, with infrequent segmental fibrinonecrotizing serositis (*n* = 1/10), intestinal nematodiasis (*n* = 1/10) or no lesions (*n* = 2/10). Viral antigen was detected mostly in the endothelium and fibroblasts and occasionally the serosa (*n* = 4/10), mostly not associated with histological lesions.

The ovary was examined from 3/10 birds, and while no histopathologic changes were seen (Figure 3g), abundant labelling was diffusely present within endothelium, cortical stroma and ovarian surface epithelium in all three birds (Figure 3h), and in the germinal epithelium of regressing follicles in 1/3 birds. The oviduct was evaluated in one bird, where a mild multifocal mononuclear salpingitis and coelomitis was evident. Abundant antigen was detected in the endothelium, tubular epithelium, and smooth muscle cells.

In the chicken examined, nonspecific histologic lesions were observed, including pyogranulomatous pneumonia, minimal focal epicarditis, minimal multifocal lymphocytic hepatitis with extramedullary haematopoiesis, minimal diffuse lymphoplasmacytic enteritis and multifocal lymphocytic serositis, and lymphoid hyperplasia. No virus antigen was detected in any of the tissues examined.

#### 3.1.4. Tissue Distribution by rRT-PCR

APMV-1 rRT-PCR analyses of tissues from necropsied pigeons revealed systemic viral distribution in all 10 pigeons, consistent with the detection of APMV-1 antigen in all examined tissues. The necropsied chicken was negative for APMV-1 in all collected tissues, indicating an absence of infection which was supported by the IHC results. All tissues were negative for AIV. Tissues were not tested for *C. psittaci*, but cloacal swabs from two necropsied pigeons were positive. All rRT-PCR results are listed in Table S6.

#### 3.1.5. Virologic and Phylogenetic Characterisation

**Figure 4:**
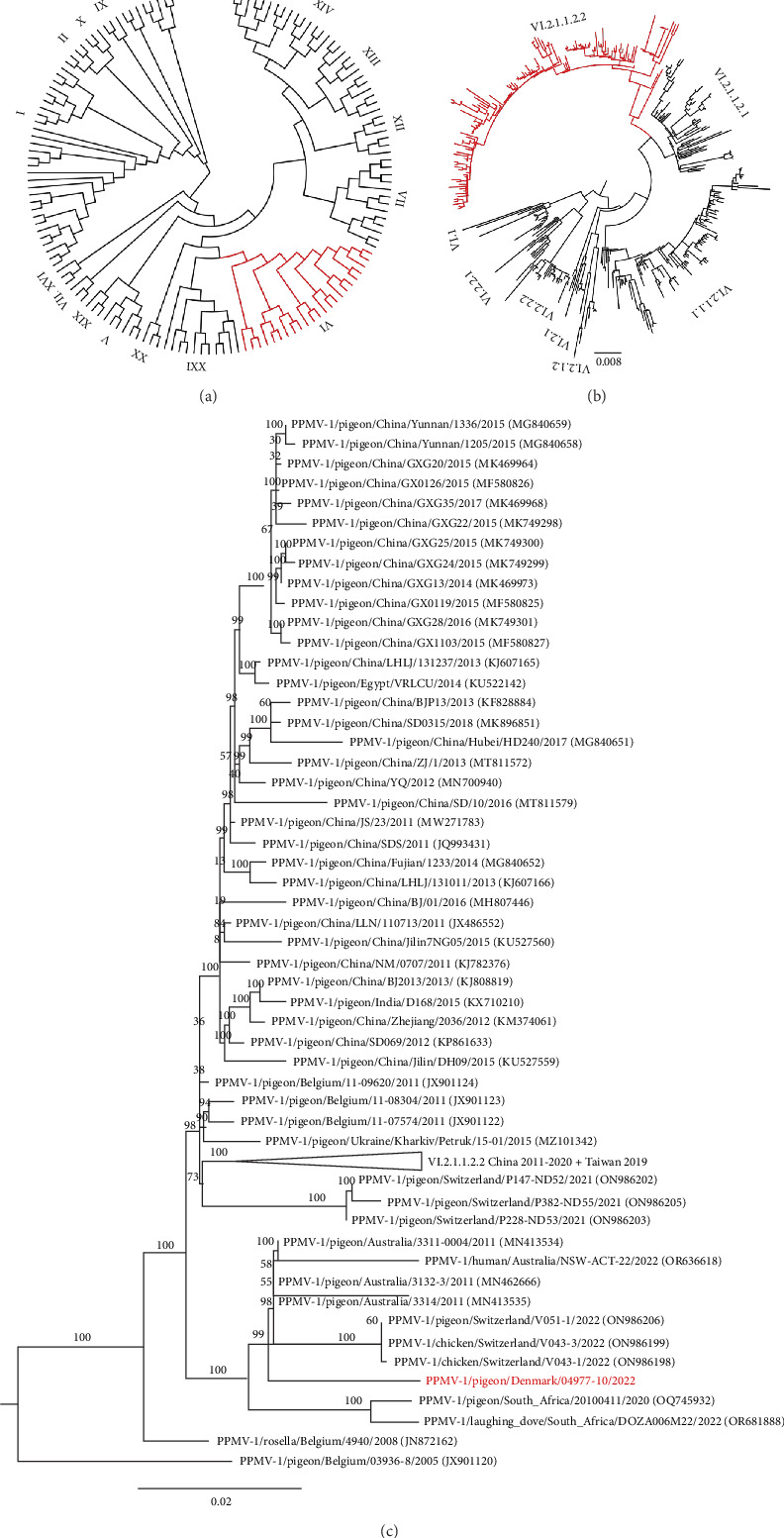
Phylogenetic analysis of the 2022 outbreak isolate PPMV-1/pigeon/Denmark/04977-10/2022 based on the complete F gene. (a) When analysed with a pilot collection of all class II genotypes, the 2022 outbreak virus clustered within genotype VI (highlighted in red). (b) Further analysis with a collection of genotype VI sequences clustered it within subgenotype VI.2.1.1.2.2 (highlighted in red). (c) Within subgenotype VI.2.1.1.2.2 the 2022 outbreak virus (red) clustered closest to Swiss, Australian and South African viruses. Branch numbers indicate bootstrap values (1000).

Sequencing of the APMV-1 isolate P/DK/04977-10/2022 F gene and genotyping by phylogenetic analysis revealed the virus to belong to Class II genotype VI.2.1.1.2.2, classifying the virus as a PPMV-1. This was supported by the reaction with monoclonal 161/617 in HI-test (Table 1). Phylogenetic analysis of the complete F gene revealed P/DK/04977-10/2022 to be closely related to viruses recently isolated in Switzerland, Australia and South Africa (Figure 4). The closest relatives to P/DK/04977-10/2022 were three isolates from Switzerland, with an average nucleotide distance of 0.03%, indicating the current isolate diverged recently from a common ancestral virus. The molecular and the in vivo pathotyping did not show a consistent profile. The molecular pathotyping revealed a polybasic cleavage site ^112^RRQKRF^117^, indicating a virulent strain, the MDT was 82.8 h indicating that the virus was mesogenic, whereas the ICPI score of 0.65 characterised the virus as lentogenic.

WGS generated a consensus sequence of 15,174 nt for P/DK/04977-10/2022. APMV-1 belonging to class II can have genome lengths of either 15,186 or 15,192 nt, with the distinguishing factor being the presence of a 6-nt insert in the 5′ non-coding region of the NP gene in genomes of 15,192 nt [46]. Since P/DK/04977-10/2022 contains this insert in its NP gene, the virus was determined to have a complete genome length of 15,192 nt. The missing 18 nt are from the 5′ trailer region, which was not sequenced.

Analysis of antigenic sites and amino acid positions associated with virulence and host adaptation revealed no notable substitutions in residues linked to decreased virulence (Table S9). One substitution linked to vaccine escape, E347G in the HN gene [42], was identified. Additionally, one substitution indicative of host adaptation to chicken was detected as P/DK/04977-10/2022 exhibits a serine (S) residue at position 431 in the NP gene, contrasting with the proline (P) found in 92.25% of pigeon-origin genotype VI viruses, as reported by Huang et al. [6]. This substitution aligns with the majority of chicken-origin viruses, where serine is present in 87.5% of genotype XX and 98.11% of genotype VII viruses.

### 3.2. Outbreak June 2023

#### 3.2.1. Pathogen Detection

APMV-1 was detected in all oropharyngeal and cloacal swabs collected from both dead, clinically sick and apparently healthy pigeons, except for one pool of cloacal swabs from clinically sick pigeons (Table S7). F gene sequencing revealed a virulent F protein cleavage site. AIV was not detected in any of the collected swab pools. APMV-1 was isolated in ECE from two pools; one pool of oropharyngeal swabs from clinically sick pigeons, one pool of cloacal swabs from dead pigeons. The virus isolated from the pool of oropharyngeal swabs collected from clinically sick pigeons was designated PPMV-1/pigeon/Denmark/05038-4/2023 (P/DK/05038-4/2023) and selected for further virologic characterisation.

#### 3.2.2. Gross Pathology

Two pigeons (a young male and an adult female) from the 2023 outbreak were necropsied. Both presented in very poor body condition and the plumage was dishevelled and matted. Pulmonic haemorrhages and consolidation were observed in the male pigeon, and hepatic congestion and pancreatic haemorrhages were seen in the adult female. Other organs were unremarkable. Ten chickens were also selected for necropsy due to their close proximity to diseased pigeons. Their overall condition, including plumage, body condition and organs were unremarkable, with the exception of two chickens exhibiting dishevelled plumage.

#### 3.2.3. Histopathology and IHC

The two necropsied pigeons were assessed by histopathology and viral IHC, but histological interpretation was limited by advanced autolysis and only small subset of tissues sampled (Figure 2). In the brain of one pigeon, mild multifocal non-suppurative encephalitis with mild lymphocytic perivascular cuffing was seen, with colocalisation of antigens in neurone and endothelium. In the lungs, diffuse pulmonary congestion was evident in one bird, with abundant viral antigen detected in the endothelium, smooth muscle and occasional macrophages, while the other showed no histological changes but a moderate amount of viral antigen in the endothelium. No lesions were identified in the trachea of both birds, but in one, minimal labelling was present in the endothelium and mucosal epithelium. Where small intestine was sampled from a single pigeon, microscopic assessment was not possible due to autolysis, but viral antigen was evident in smooth muscle and endothelium of the intestinal wall.

#### 3.2.4. Tissue Distribution by rRT-PCR

All tissues collected from necropsied pigeons were positive by rRT-PCR for APMV-1, consistent with systemic distribution (Table S8). This widespread dissemination was further supported by the detection of antigen by IHC in the brain of one pigeon. In contrast, APMV-1 was not detected in oropharyngeal and cloacal swabs or tissues from the necropsied chickens.

#### 3.2.5. Virologic and Phylogenetic Characterisation

**Figure 5:**
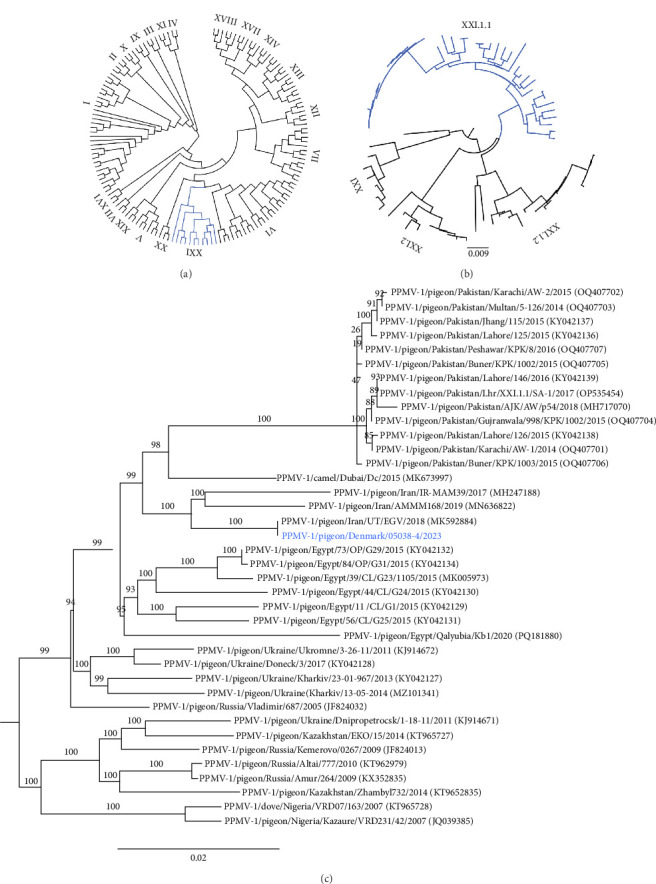
Phylogenetic analysis of the 2023 outbreak isolate PPMV-1/pigeon/Denmark/05058-4/2023 based on the complete F gene. (a) When analysed with a pilot collection of all class II genotypes, the 2023 outbreak virus clustered within genotype XXI (highlighted in blue). (b) Further analysis with a collection of genotype XXI viruses clustered it within subgenotype XXI.1.1 (highlighted in blue). (c) Within subgenotype XXI.1.1 the 2023 outbreak virus (blue) clustered closest to recently isolated Iranian viruses. Branch numbers indicate bootstrap values (1000).

Genetic and phylogenetic analysis of the isolate P/DK/05038-4/2023 F gene showed the virus to belong to Class II genotype XXI.1.1, classifying it as a PPMV-1. Complete F gene phylogenetic analysis showed the 2023 outbreak virus to be closely related to viruses from Iran, Dubai, Egypt and Pakistan (Figure 5). The closest relative to P/DK/05038-4/2023 was an isolate from Iran, with a nucleotide distance of only 0.02%. The cleavage site was polybasic with the motif ^112^KRQKRF^117^ indicative of a virulent pathotype. The MDT was 72 h indicative of a mesogenic pathotype. An ICPI test was not performed. Genetic and pathogenic properties of the viruses from both the 2022 and 2023 outbreaks are summarised in Table 2. HI tests with an APMV-1 (LaSota) and PPMV-1 resulted in HI titres of 128 and 16, respectively. HI tests with monoclonal antibodies U85, 161/617 and 7D4 were all negative (Table 1).

Since P/DK/05038-4/2023 belongs to class II, the full genome can be either 15,186 or 15,192 nt [46]. WGS resulted in a sequence of 15,192 nt, consisting of the full coding sequences of all six genes. Interestingly, the start codon of the F gene was identified as ACG instead of the canonical ATG. This finding was confirmed through both WGS and targeted F gene sequencing, which were performed independently. Only the F gene exhibited this unusual start codon.

WGS analysis of P/DK/05038-4/2023 for antigenic sites and amino acid positions associated with virulence and host adaptation revealed no significant substitutions linked to decreased virulence (Table S9). However, two substitutions indicative of adaptation to chickens were identified in the F gene: I50V and H259Q. Typically, isoleucine (I) at position 50 and histidine (H) at position 259 are present in 97.67% and 93.01% of pigeon-origin genotype VI viruses, respectively, as reported by Huang et al. [6]. Additionally, three substitutions indicative of increased virulence were identified in the HN gene: N263K, V266A and I514V. Positions 253 and 514 are located in a neutralising epitope of the HN gene and have been associated with neutralising vaccine escape variants [42]. Position 266 has been reported to be under positive selection in the HN gene to be located in a potential B cell epitope [47].

## 4. Discussion

This study describes two separate PPMV-1 outbreaks in Denmark. In June 2022, an outbreak occurred in Stenbæksholm and Stenstrup, southeast Denmark, involving a genotype VI.2.1.1.2.2 PPMV-1 (P/DK/04977-10/2022). One year later, in June 2023, another outbreak occurred in Aalborg, northwest Denmark, involving a genotype XXI.1.1 PPMV-1 (P/DK/05038-4/2023). The separation in time, region and genotype documents that these outbreaks were unrelated and show that different pigeon-specific genotypes are occurring in Denmark.

In both outbreaks, clinical signs were consistent with PPMV-1 infection and included high mortality and severe neurological symptoms in some pigeons, while others exhibited mild depression. The 2022 outbreak affected 851 pigeons with variable mortality (25%–100%), whereas the 2023 outbreak involved 40 pigeons, resulting in 30 deaths. In chickens, mild clinical signs such as increased hatching time and embryonic mortality were observed in 2022, but no clinical signs from chicken were reported in the 2023 outbreak. Ten chickens from the 2023 outbreak were necropsied and tested for APMV-1 in oropharyngeal and cloacal swabs. None showed pathology consistent with APMV-1 infection or tested positive for APMV-1, including one chicken found dead, suggesting its death was unrelated to APMV-1. Pigeons from the 2022 outbreak had reportedly been sick for an extended period (weeks to months), with some birds recovering and later exhibiting clinical signs again. However, only three out of the 11 pigeons, from which blood was collected, tested positive for antibodies against APMV-1. This suggests that the pigeons may not have been infected by APMV-1 for an extended period, as that would have resulted in a higher proportion of antibody-positive birds. Additionally, concurrent or secondary bacterial and mycotic infections were present, as *C. psittaci* was detected in some birds, and bacterial colonies and fungi were found in the lungs of four necropsied pigeons. Together with variations in vaccination status, these factors complicate the assessment of viral virulence based on clinical signs and pathology.

Despite concurrency of secondary infections in some birds, the consistent clinical picture, and characteristic pathological findings across birds with tandem association of virus in situ and inflammation in multiple organ systems, particularly frequent viral-associated non-suppurative encephalitis, strongly support PPMV-1 as the primary aetiological agent for the demise of the birds. While secondary infections may have contributed to morbidity they are unlikely to account for the outbreak pattern and high mortality. Although fulfilling Koch's postulates would require experimental infection studies in pigeons, such approach is limited by ethical and practical constraints. As such, causation in these outbreaks is therefore inferred from the consistent convergence of pathological and virological findings.

Microscopic analyses of pigeons from both outbreaks demonstrated systemic viral antigen distribution with multi-cellular tropism, including neuronal and vascular endothelium. In the 2022 outbreak, non-suppurative meningoencephalitis and tubulointerstitial nephritis were the most common findings, while the 2023 outbreak, though limited to two pigeons, included one case of viral-associated encephalitis. These microscopic lesions are similar to those natural disease outbreaks associated with pathogenic PPMV-1 in doves and pigeons reported elsewhere [48–50]. Additionally, viral antigen detected in reproductive tissues from the 2022 outbreak suggests a potential relationship with impaired egg production and thin-shelled eggs observed during the outbreak. Overall, the consistent systemic presence of viral antigen, combined with acute disease-causing high morbidity and mortality, demonstrates virulent phenotypes of PPMV-1 from the captive pigeons in this study.

APMV-1 is a zoonotic pathogen, occasionally causing mild conjunctivitis in humans [51]. There have, however, been reports of fatal cases in immunosuppressed individuals infected with PPMV-1. For instance, a genotype VI.2.1.1.2.2 virus (Figure 4, acc. no. OR636618) closely related to P/DK/04977-10/2022 caused fatal respiratory disease in an immunosuppressed patient in Australia in 2022 [52]. Similar fatal cases include pneumonia in immunosuppressed individuals following stem cell transplants [53, 54] and encephalitis caused by a genotype XXI virus in an immunodeficient child [55]. These cases highlight the risk associated with close contact and handling of pigeons, particularly for immunosuppressed individuals, and suggest that screening for and vaccination of pigeons against PPMV-1 could be beneficial in environments where pigeon flocks are routinely handled, such as the affected allotment house communities described in this study.

P/DK/04977-10/2022 and P/DK/05038-4/2023 were both classified as PPMV-1, as they belonged to the pigeon-associated genotypes VI and XXI, were isolated from pigeons, and demonstrated apparent host adaptation to pigeons by primarily causing disease in this host. They were tested against monoclonal antibody 161/617, which is commonly used to differentiate PPMV-1 from APMV-1 [25]. However, P/DK/05038-4/2023 did not react to this antibody, nor to other monoclonal antibodies, such as 7D4 and U85, which typically react with non-pigeon APMV-1 strains [26]. Cases of PPMV-1 strains failing to react with the 161/617 monoclonal antibody have been reported previously [56]. Given that genotype XXI evolved from genotype VI, and that 161/617 was developed based on genotype VI, this lack of reactivity might reflect antigenic changes resulting from this evolutionary divergence. The F protein cleavage site, a key determinant of APMV-1 pathogenicity, varied between the two viruses. P/DK/04977-10/2022 possessed the ^112^RRQKRF^117^ motif, which has been reported as the most dominant motif in virulent APMV-1 viruses and is found in the majority of class II genotypes [57]. P/DK/05038-4/2023 exhibited the rarer virulent ^112^KRQKRF^117^ motif, which, under the older nomenclature used in [57], was reported in genotype V-VII. Using the updated nomenclature as clarified by [5], this now includes genotypes V-VII, XX, XIX and XXI.

Molecular pathotyping classified both viruses as virulent due to their polybasic cleavage sites. However, there were discrepancies with the biological tests of ICPI and MDT. P/DK/04977-10/2022 was classified as lentogenic by ICPI score (0.65) and mesogenic by MDT (82.8 h), aligning with the limited clinical impact in chickens, but contrasting with the high mortality in pigeons. Similarly, P/DK/05038-4/2023 was classified as mesogenic by MDT (72 h) despite causing severe disease in pigeons. Contrasting pathotyping results are often seen with PPMV-1 viruses, as they frequently possess virulent cleavage sites but exhibit varying ICPI and MDT results [6, 58–61]. These inconsistencies in pathotyping methods suggests that that reliance on the F gene cleavage site as a sole molecular marker for PPMV-1 classification may be insufficient [62] and highlight the limitations of using chicken tests for assessing PPMV-1 pathogenicity in other species such as pigeons. Experimental infection studies in cognate host species and using natural routes could provide more accurate assessments of pathogenicity in non-chicken hosts [22].

P/DK/04977-10/2022 clustered closely with PPMV-1 isolates from a 2011 outbreak in Australian racing pigeons (Figure 4), which, like the Danish isolate, exhibited a polybasic cleavage site, an MDT indicative of a mesogenic pathotype, caused high pigeon mortality, but did not induce clinical signs in experimentally infected chickens [63]. However, the Australian study found that infected chickens shed the virus asymptomatically, raising the question whether chickens in the Danish 2022 outbreak also acted as subclinical shedders. Only one chicken from the 2022 outbreak was tested for APMV-1 and found negative by IHC and rRT-PCR, despite descriptions of chickens displaying mild clinical signs during the outbreak period. Since P/DK/04977-10/2022 and the Australian isolates were only compared phylogenetically based on their F genes, their true similarity remains unknown. Consequently, the potential of the Danish viruses for transmitting to chickens is also unknown, as virulence and host adaptation are also influenced by factors beyond the F gene [6, 64–66].

Besides the Australian isolates mentioned before, P/DK/04977-10/2022 also clustered closely with strains from an outbreak in Switzerland in January 2022 [10], where PPMV-1 was isolated from both feral pigeons and chickens (Figure 4). In both outbreaks, chickens exhibited minimal clinical signs but were affected in their laying cycles. In Switzerland, decreased egg production was noted, while in Denmark, chickens experienced prolonged hatching times and hatching of dead chicks. Unlike the Swiss pigeons, which appeared healthy, Danish pigeons suffered severe clinical outcomes, likely due to differences in exposure history. PPMV-1 was found to be endemic in the Swiss feral pigeons [10], whereas the Danish fancy and racing pigeons had no known history of PPMV-1 infections. Although chickens were not severely affected during these outbreaks, allowing the virus to circulate further could have increased its pathogenicity and posed a risk to nearby poultry farms, as PPMV-1 virulence in chickens has been known to increase with successive passages [67–69,[70]. Prompt culling and biosecurity measures such as protection zones are therefore essential to limit the potential for viral host adaptation.

Regions of the F gene, along with additional genes such as HN, NP, P and L, play significant roles in modulating virulence and host adaptation [6, 64–66]. Mutations in the L, P and NP genes have been associated with enhanced replication efficiency in chickens, suggesting that the viral replication complex plays a significant role in the evolutionary adaptation of PPMV-1 from pigeons to chickens [6, 65]. The analysis of P/DK/04977-10/2022 identified the substitution P431S in the NP gene, and analysis of P/DK/05038-4/2023 identified the substitutions I50V and H259Q in the F gene, which are all mutations commonly found in chicken-origin viruses. However, 36 of such mutations have been identified [6], so the limited number of host-adaptation substitutions in the two Danish isolates suggests that the two viruses have not undergone extensive adaptation to chickens. This is further supported by the minimal to absent clinical signs observed in chickens during the outbreaks and the absence of virus detection by IHC and rRT-PCR in the necropsied chickens. The substitutions might instead be the result of passaging in ECEs during virus isolation, as PPMV-1s have been known to mutate during passaging [65, 71]. In P/DK/04977-10/2022, the HN gene exhibited the substitution E347G, while in P/DK/05038-4/2023, the substitutions N263K, V266A and I514V were identified. Substitutions E347G, N263K and I514V have been associated with vaccine escape [42], indicating a role in immune evasion. Similarly, V266A, a predicted B-cell epitope linked to positive selection pressure [47], further supports the involvement of immune-driven selection in the evolution of these viruses. However, the varied vaccination statuses of the pigeons involved in the outbreaks raise important questions about the selective pressures driving the emergence of these substitutions.

The start codon of the P/DK/05038-4/2023 F gene was identified as ACG instead of the canonical ATG, confirmed by both WGS using the Ion Torrent platform and F gene sequencing with Illumina. Non-canonical start codons, including ACG, CTA and ATA, have been previously described in class I APMV-1 strains. Importantly, strains with the ACG start codon have been shown to retain virulence and replication efficiency comparable to those with the canonical ATG, while ATA has been associated with increased virulence [72]. The presence of the ACG start codon is not unique to APMV-1 but has also been reported in other paramyxoviruses, such as the Sendai virus, where it functions effectively as a translational start codon [73]. These observations suggest that the non-canonical ACG start codon in the P/DK/05038-4/2023 F gene is functional and is unlikely to significantly impact viral fitness.

Phylogenetic analysis revealed that P/DK/055038-4/2023 clustered closely with isolates from Iran (Figure 5), where genotype XXI.1.1 has been circulating since the early 2010s. The recent detection of genotype VI.2.1.2 in Iran further demonstrates that genotypes XXI and VI co-circulate not only in Denmark, but also in other parts of the world [60]. Additionally, a recent study on PPMV-1 epidemiology and temporal distribution reported an increase in genotype XXI detections during the 2010s, with this genotype predominantly found in Pakistan, Bangladesh, Egypt and Russia, while genotype VI continues to be distributed worldwide [6].

The global spread of PPMV-1 has often been linked to human activities, such as pigeon transportation, trade and exhibitions, as pigeons are non-migratory and unlikely to disperse viruses over long distances independently [74, 75]. Notably, the F gene of P/DK/05038-4/2023 showed a nucleotide distance of only 0.02% to an Iranian isolate, while P/DK/04977-10/2022 differed by just 0.03% from Swiss isolates. These minimal genetic distances indicate recent shared ancestry and likely reflect human-mediated transmission across regions. However, the absence of surveillance in Danish feral pigeons means that undetected local or regional transmission pathways cannot be excluded. Further, studies from São Paulo and Zurich, have identified feral pigeon populations as significant reservoirs of PPMV-1, and as such there is possibility of previously unrecognised urban reservoirs [10, 76]. The Swiss isolates closely related to P/DK/04977-10/2022 were detected in feral pigeons outside Zurich [10], suggesting the Danish outbreaks may likewise have emerged from undetected local reservoirs potentially introduced from other regions via wild birds. Moreover, resident wild birds have been shown to act as bridge hosts, transmitting PPMV-1 across ecological boundaries and into migratory bird populations that spread the virus over larger distances and reseed it into new feral pigeon populations [75], which should also be examined in the future.

In both Danish outbreaks, no definitive source was identified, and no other pigeon populations were reported as affected or under suspicion at the time. It, therefore, remains unclear whether these viruses were introduced through human activity or via spill-over from wild birds such as feral pigeons. The circulation and genetic diversity of PPMV-1 in feral Danish birds remain unexplored. Determining whether these viruses are present and identifying their genotypes could provide valuable insights into and risk recognition of the origin of the outbreak viruses, their transmission pathways, and the broader epidemiology of avian populations in Denmark.

## Figures and Tables

**Table 1:** HI reactivity of polyclonal antisera and monoclonal antibodies.

Isolates	Polyclonal antisera		Monoclonal antibodies		
	LaSota^a^	PPMV-1^b^	U85	161/617	7D4
P/DK/04977-10/2022	128	64	<8	256	<8
P/DK/05038-4/2023	128	16	<8	<8	<8

^a^Class II genotype II.

^b^Class II genotype VI.1.

**Table 2:** Genetic and pathogenic properties of outbreak viruses.

Isolates	F gene cleavage site	Genotype (Class II)	MDT^a^ (h)	ICPI^b^
P/DK/04977-10/2022	^112^RRQKRF^117^	VI.2.1.1.2.2	82.8	0.65
P/DK/05038-4/2023	^112^KRQKRF^117^	XXI.1.1	72	n/a

*Note*: n/a, analysis not done.

^a^MDT < 60 h is categorised as velogenic, 60–90 h as mesogenic and >90 h as lentogenic.

^b^ICPI > 1.5 is categorised as velogenic, 0.7–1.5 as mesogenic and <0.7 as lentogenic.

## Data Availability

The consensus sequences generated in this study have been submitted to the GenBank database under accession numbers PV260999 and PV261000.

## Supporting Information

Supporting Information Tables S1 and S2 list the BLAST reference sequences used for phylogenetic analyses of the 2022 and 2023 outbreak isolates. Tables S3–S6 present rRT-PCR results from swab pools and necropsied birds, as well as HI data from blood samples collected during the 2022 outbreak. Tables S7 and S8 contain rRT-PCR results from the 2023 outbreak. Table S9 summarises amino acid changes identified in the genomes of the outbreak viruses associated with virulence, host adaptation and known antigenic sites.
