# Supplementary material for: Two Outbreaks of Pigeon Paramyxovirus 1 With High Mortality in Captive Pigeons (Columbia livia) in Denmark, 2022–2023
Source: Transbound Emerg Dis. 2025 Sep 29;2025:5629889. doi: 10.1155/tbed/5629889 (PMC12500374; doi:10.1155/tbed/5629889)
Supplement: Supporting Information — Tables S1 and S2 list the BLAST reference sequences used for phylogenetic analyses of the 2022 and 2023 outbreak isolates. Tables S3–S6 present rRT-PCR results from swab pools and necropsied birds, as well as HI data from blood samples collected during the 2022 outbreak. Tables S7 and S8 contain rRT-PCR results from the 2023 outbreak. Table S9 summarises amino acid changes identified in the genomes of the outbreak viruses associated with virulence, host adaptation and known antigenic sites. [file 5629889.f1.pdf]

Transboundary and Emerging Diseases (2025)

Two outbreaks of pigeon paramyxovirus 1 with high mortality in captive pigeons (Columbia livia) in Denmark, 2022-2023

Karen Martiny, Audra-Lynne D. Schlachter, Tim K. Jensen, Fabian Z. X. Lean, Alejandro Núñez, Anne Sofie Hammer, Christian Grund, Dirk Höper, Solvej Ø. Breum, Jens P. Christensen, Lars E. Larsen and Charlotte K. Hjulsager

Supplementary Materials

TABLE S1. BLAST sequences (genotype VI.2.1.1.2.2) included in phylogenetic analysis of 2022 outbreak isolate

Designation*	Accession no.	Host Species	Country	Year
PPMV-1/pigeon/Australia/3314/2011	MN413535	Pigeon	Australia	2011
PPMV-1/pigeon/China/SH/CH/041002/2012	PP297102	Pigeon	China	2011
PPMV-1/pigeon/China/SH/CH/050201/2012	PP297098	Pigeon	China	2012
PPMV-1/pigeon/China/SH/CH/051401/2012	PP297097	Pigeon	China	2012
PPMV-1/pigeon/China/SH/CH/0168/2013	KT163263	Pigeon	China	2013
PPMV-1/pigeon/China/SH/CH/163/2013	KJ600778	Pigeon	China	2013
PPMV-1/pigeon/Heilongjiang/BS1/2018	OR253509	Pigeon	China	2018
PPMV-1/pigeon/South_Africa/20100411/2020	OQ745932	Pigeon	South Africa	2020
PPMV-1/pigeon/Switzerland/P147-ND52/2021	ON986202	Pigeon	Switzerland	2021
PPMV-1/pigeon/Switzerland/P228-ND53/2021	ON986203	Pigeon	Switzerland	2021
PPMV-1/pigeon/Switzerland/P382-ND55/2021	ON986205	Pigeon	Switzerland	2021
PPMV-1/chicken/Switzerland/V043-1/2022	ON986198	Chicken	Switzerland	2022
PPMV-1/chicken/Switzerland/V043-3/2022	ON986199	Chicken	Switzerland	2022
PPMV-1/pigeon/Switzerland/V051-1/2022	ON986206	Pigeon	Switzerland	2022
PPMV-1/human/Australia/NSW-ACT-22/2022	OR636618	Human	Australia	2022
PPMV-1/laughing_dove/South_Africa/DOZA006M22/2022	OR681888	Laughing dove	South Africa	2022

*Designation used in phylogenetic tree in Figure 4

TABLE S2. BLAST sequences (genotype XXI.1.1) included in phylogenetic analysis of 2023 outbreak isolate

Designation*	Accession no.	Host Species	Country	Year
PPMV-1/pigeon/Pakistan/Karachi/AW-1/2014	OQ407701	Pigeon	Pakistan	2014
PPMV-1/pigeon/Pakistan/Multan/5-126/2014	OQ407703	Pigeon	Pakistan	2014
PPMV-1/pigeon/Pakistan/Karachi/AW-2/2015	OQ407702	Pigeon	Pakistan	2015
PPMV-1/pigeon/Pakistan/Gujranwala/998/KPK/1002/2015	OQ407704	Pigeon	Pakistan	2015
PPMV-1/pigeon/Pakistan/Buner/KPK/1002/2015	OQ407705	Pigeon	Pakistan	2015
PPMV-1/pigeon/Pakistan/Buner/KPK/1003/2015	OQ407706	Pigeon	Pakistan	2015
PPMV-1/pigeon/Pakistan/Peshawar/KPK/8/2016	OQ407707	Pigeon	Pakistan	2016
PPMV-1/pigeon/Pakistan/Lhr/XXI.1.1/SA-1/2017	OP535454	Pigeon	Pakistan	2017
PPMV-1/pigeon/Egypt/Qalyubia/Kb1/2020	PQ181880	Pigeon	Egypt	2020

*Designation used in phylogenetic tree in Figure 5

TABLE S3. 2022 outbreak. real-time reverse-transcriptase PCR (rRT-PCR) results for avian paramyxovirus 1 (APMV-1) of swab pools

		rRT-PCR assay		
	**Pool[1]**	**Large polymerase (L) gene[2]** (FAM | HEX)	**Matrix (M) gene**	**Fusion (F) gene**
**Oropharyngeal swabs**	1	32.78 | 36.75	44.89	neg
	2	neg |neg	neg	neg
	3	39 | neg	neg	neg
	4	neg | neg	neg	neg
	5	23.37 | 26.64	28.48	pos
**Cloacal swabs**	1	29.39 | 32.54	37.77	pos
	2	39.9 | neg	neg	neg
	3	35.07 | 38.72	neg	neg
	4	38.68 | neg	neg	neg
	5	23.05 | 26.4	28.08	pos

[1]All pools consist of 5 swabs. Pool 1-4 from live pigeons, and pool 5 from dead pigeons.

[2]The L-gene assay consists of two probes (FAM and HEX)

[3]The result of this PCR assay are pos/neg as they are determined by the presence or absence of specific DNA bands following gel electrophoresis.

neg = negative, pos = positive

TABLE S4. 2022 outbreak. rRT-PCR results for *Chlamydia psittaci* of swab pools

	rRT-PCR	
**Pool**	**OP swabs**	**Cloacal swabs**
**1**	neg	41.07
**2**	neg	neg
**3**	neg	neg
**4**	neg	neg
**5**	neg	neg
**6**	neg	neg
**7**	neg	37.5
**8**	neg	neg
**9**	neg	neg
**10**	neg	neg

OP = oropharyngeal

All pools consist of 3 swabs

TABLE S5. 2022 outbreak. Hemagglutination inhibition (HI) titres of blood samples from live pigeons[1]

Pigeon	Virus/Viral antigen		
**#**	**P/DK/04977-10/2022**	**PPMV-1**	**APMV-1 (LaSota)**
**1**	<16	<16	<16
**2**	1024	2048	512
**3**	<16	<16	<16
**4**	<16	<16	<16
**5**	<16	<16	<16
**6**	<16	<16	<16
**7**	512	1024	512
**8**	<16	<16	<16
**9**	<16	<16	<16
**10**	<16	<16	<16
**11**	512	512	256

<16 = negative

[1]These are not the same pigeons as the necropsied pigeons

TABLE S6. 2022 outbreak. rRT-PCR results for APMV-1 and *Chlamydia psittaci* of necropsied pigeons and chicken

Bird	Age	Sex	Large polymerase (L) gene (APMV-1)												Ornithosis
			Cloacal swab	Organs											Cloacal swab
				B	H	Lu	T	Li	P	D	K	S	Ct	Cl	
**P1**	a	m	20.91	27.08	29.58	20.69	24.59	29.01	n/a	24.55	21.16	22.06	22.53	16.29	neg
**P2**	a	f	16.79	24.42	20.73	19.49	17.32	22.08	15.85	15.42	14.28	16.63	16.92	19.89	neg
**P3**	a	m	22.48	29.53	33.84	26.49	29.56	31.06	19.61	26.85	20.12	n/a	25.6	21.02	neg
**P4**	a	f	18.23	28.26	23.47	20.9	20.41	18.25	18.02	17.6	18.17	16.84	17.1	19.31	neg
**P5**	a	f	19.18	24.39	27.77	24.03	21.52	32.32	19.05	23.17	16.37	n/a	n/a	n/a	39.6
**P6**	a	m	20.68	27.53	24.92	24.11	22.65	27.12	17.31	22.13	19.02	n/a	n/a	n/a	22.4
**P7**	a	f	19.89	25.6	23.77	20.33	18.48	23.49	14.76	17.4	15.53	n/a	n/a	n/a	neg
**P8**	a	m	22.28	29.13	29.5	28.89	25.08	32.1	25.2	24.93	18.4	n/a	n/a	n/a	neg
**P9**	a	f	26.55	31.3	28.37	27.24	34.74	33.35	26.19	26.52	25.64	n/a	n/a	n/a	neg
**P10**	a	f	35.54	36.67	neg	31.71	34.93	neg	35.05	32.8	37.25	n/a	n/a	n/a	neg
**Ck**	a	f	neg	neg	neg	neg	neg	neg	neg	neg	neg	neg	neg	n/a	neg

a = adult, j = juvenile, m = male, f = female, B = brain, H = heart, Lu = lung, T = trachea, Li = liver, P = pancreas, D = duodenum, K = kidney, S = spleen, Ct = caecal tonsil, Cl = cloaca, n/a = not analysed, neg = negative

TABLE S7. 2023 outbreak. rRT-PCR results for APMV-1 of swab pools

	Pool	No. swabs[1]	**rRT-PCR assay**		
			**Large polymerase (L) gene[2]** (FAM | HEX)	**Matrix (M) gene**	**Fusion (F) gene[3]**
**Oropharyngeal swabs**	1	5	37,38 | 39,43	neg	neg
	2	5	32,98 | 34,93	34,46	pos
	3	5	32,99 | 34,82	34,00	pos
	4	4	25,25 | 27,07	26,5	pos
	5	2	25,4 | 27,22	27.4	pos
**Cloacal swabs**	1	5	neg | neg	neg	neg
	2	5	30,04 | 32,07	30,81	pos
	3	5	28,34 | 30,28	29,36	pos
	4	4	27,75 | 29,73	29,24	pos
	5	2	25,7 | 27,58	27.48	pos

[1]All pools consist of 2-5 swabs. Pool 1-3 from symptomatic pigeons, pool 4 from asymptomatic pigeons, and pool 5 from two dead pigeons.

[2]The L-gene assay consists of two probes (FAM and HEX)

[3]The result of this PCR assay are pos/neg as they are determined by the presence or absence of specific DNA bands following gel electrophoresis.

neg = negative, pos = positive

TABLE S8. 2023 outbreak. rRT-PCR results for APMV-1 in two necropsied pigeons

rRT-PCR assay[1]	Swabs				Organs from P1 and P2 in pools				
	**OP – P1**	**CL – P1**	**OP – P2**	**CL – P2**	**T**	**B**	**Lu**	**In**	**Ct**
**L (FAM)**	28.07	24.78	29.23	24.97	25.43	22.82	22.03	22.64	23.04
**L (HEX)**	28.73	25.38	30.09	25.58	26.26	23.55	22.83	23.42	23.84
**M**	28.14	23.26	28.51	23.71	24.48	23.13	22.39	21.71	24.58
**F[3]**	pos	pos	pos	pos	pos	pos	pos	pos	pos

P1 = pigeon 1 (juvenile, male)

P2 = pigeon 2 (adult, female)

OP = oropharyngeal swab, CL = cloacal swab, T = trachea, B = brain, Lu = lung, In = intestine, Ct = caecal tonsil

[1]The L-gene assay consists of two probes (FAM and HEX)

[3]The result of this PCR assay are pos/neg as they are determined by the presence or absence of specific DNA bands following gel electrophoresis.

TABLE S9 Antigenic sites and amino acid positions associated with virulence and host adaptation

			Amino acid residue		
**Function**	**Gene**	**Position**	**P/DK/04977-10/2022**	**P/DK/05038-4/2023**	**Reference**
Host adaptation (pigeon to chicken)	NP	P431S	S	P	(Huang et al., 2024)[A]
	P	N37D	S	S	(Dortmans et al., 2011b)
	F	I50V	I	V	(Huang et al., 2024)[A]
		H259Q	H	Q	(Huang et al., 2024)[A]
	L	N1564S	N	T	(Dortmans et al., 2011b)
		V1694E	V	V	(Dortmans et al., 2011b)
Increases virulence	F	K78R	K	R	(Umali et al., 2014)
	HN	D115S	N	N	(Mast et al., 2006)
		L229R	L	L	(Mast et al., 2006)
		N263K	L	K	(Umali et al., 2014)
		V266A	T	A	(Gu et al., 2011)
		E347K	G	E	(Cho et al., 2008; Gu et al., 2011; Hu et al., 2010)
		E347Q	G	E	(Mayahi and Esmaelizad, 2017)
		E347G	G	E	(Umali et al., 2014)
		G362K	G	G	(Mayahi and Esmaelizad, 2017)
		I514V	F	V	(Umali et al., 2014)
Decreases virulence	M	R42A	R	R	(Duan et al., 2014)
		G275A	G	G	(Xu et al., 2016)
		P276A	P	P	(Xu et al., 2016)
	F	D72Y	D	D	(Mast et al., 2006)
		R101M	R	R	(Mast et al., 2006)
		Q114R	Q	Q	(Samal et al., 2011)
		S453P	S	S	(Jos C.F.M. Dortmans et al., 2010)
	L	K1756A	E	K	(Ji et al., 2018)
		D1881A	D	D	(Ji et al., 2018)
		K1917A	K	K	(Ji et al., 2018)
		E1954Q	E	E	(Ji et al., 2018)

[A]In this reference, 36 host-specific amino acid sites in PPMV-1 genotype VI were compared to chicken-origin APMV-1s. Only the positions where the outbreak viruses exhibited the chicken adapted amino acids are shown in this table.
